# Analysis of Autoantibodies against Promyelocytic Leukemia Nuclear Body Components and Biochemical Parameters in Sera of Patients with Primary Biliary Cholangitis

**DOI:** 10.3390/diagnostics11040587

**Published:** 2021-03-24

**Authors:** Alicja Bauer, Andrzej Habior, Paulina Wieszczy, Damian Gawel

**Affiliations:** 1 Department of Biochemistry and Molecular Biology, Centre of Postgraduate Medical Education, 01-813 Warsaw, Poland; damian.gawel@cmkp.edu.pl; 2 Department of Gastroenterology, Hepatology and Clinical Oncology, Centre of Postgraduate Medical Education, 02-781 Warsaw, Poland; ahab@coi.waw.pl (A.H.); pwieszczy@cmkp.edu.pl (P.W.); 3 Clinic of Polish Gastroenterology Foundation, 02-653 Warsaw, Poland; 4 Department of Immunohematology, Centre of Postgraduate Medical Education, 01-813 Warsaw, Poland

**Keywords:** autoantibodies, biochemical parameters, liver, primary biliary cholangitis, promyelocytic leukemia nuclear body components

## Abstract

Primary biliary cholangitis (PBC) is a chronic autoimmune liver disease characterized by immune-mediated destruction of intrahepatic bile ducts and the presence of specific antibodies. The aim of the study was to examine the diagnostic significance of antibodies against promyelocytic leukemia nuclear body (PML NB) components such as Sp100, Sp140, and PML in a cohort of PBC patients and compare the results with biochemical and histological parameters. Serum samples were collected from 93 PBC patients. Anti-Sp100 and anti-PML antibodies were assessed using commercially available kits, anti-Sp140 using developed “in-house” ELISA test. Anti-Sp140, anti-Sp100, and anti-PML antibodies were present in 25 (27%), 37 (40%), and 29 (31%) PBC patients, respectively. Anti-PML NB positive patients also showed increased concentration of bilirubin and alkaline phosphatase (*p* < 0.05). In the group with the presence of at least two types of these antibodies, more frequent deaths or transplantations were observed. A correlation between the presence of antibodies and histological grade (OR = 2.55 *p* = 0.039) was established. Patients with bilirubin > 1.1 mg/dL at the time of diagnosis had a significantly shorter time of survival than patients with bilirubin ≤ 1.1 mg/dL (HR 5.7; 95% C.I., 2.7, 12.3; *p* < 0.001). Our data confirm very high specificity of anti-PML NB antibodies, which can expand the laboratory diagnostic capabilities of PBC. We found an association between positive reactivity of autoantibodies directed against components of PML nuclear bodies and higher concentrations of bilirubin and alkaline phosphatase, but the main prognostic marker of survival remains serum bilirubin.

## 1. Introduction

Primary biliary cholangitis (PBC) is a chronic, slowly progressive cholestatic liver disease, which is histologically characterized by portal inflammation and immune-mediated damage of the intrahepatic bile ducts [1,2,3,4]. Manifestation of several autoimmune features in patients with PBC has led to the generally accepted concept that the disease has an autoimmune pathogenesis [4,5,6]. PBC patients have autoantibodies directed against a variety of cellular components. Anti-mitochondrial antibodies (AMAs) are the most characteristic immunological feature of this entity [4,7]. Serum AMAs are present in more than 90% of PBC patients and 40–50% of PBC patients also show antinuclear antibodies (ANAs) [8,9,10,11,12]. The ANA pattern in PBC includes multiple nuclear dots (MND-ANA) and nuclear membrane-associated ANAs (NM-ANAs). About 30% of patients manifest anti-nuclear envelope (NE) positivity [13,14,15,16]. In the majority of cases, anti-NM antibodies target gp210 and p62 proteins. The MND pattern specific for PBC is the result of antibodies directed against structural components of promyelocytic leukemia protein nuclear bodies-PML NBs [17,18,19,20]. Among the numerous substructures of the cell nucleus, PML NBs containing PML and Sp100 proteins have attracted the interest of many researchers in recent years [17,21]. Nevertheless, the mechanisms leading to the production of autoantibodies in PBC have still not been explained.

The Sp100 and PML proteins act as transcriptional regulators. Sp100 is localized to punctate domains in the nucleus nuclear dots or nuclear bodies. PML was originally identified as a protein aberrantly expressed in leukemic cells of patients with acute promyelocytic leukemia. Based on different studies, anti-Sp100 and anti-PML autoantibodies can be detected in 20–30% of patients with PBC [7,19,20,21,22,23,24,25,26,27]. The co-localization of Sp100 and PML in nuclear dots suggests association of both proteins in multi-subunit complexes. ANAs are a relevant tool for diagnosis of PBC, especially in AMA-negative patients, and their high specificity for PBC has been confirmed in several reports [22,28,29]. The existence of anti-Sp100 antibodies in PBC patients was related with a negative course of the disease [8]. Sp140L is the phylogenetically closest member of the anti-sp100 protein family and is an autologous antigen in PBC patients [30]. Sp140 was identified by Granito and co-workers as a PML NB component and characterized using serum from patients with PBC [31]. Although PBC patients’ serum was initially used to identify Sp140, the prevalence and clinical significance of anti-Sp140 antibodies in PBC is still unknown.

The diagnostic and clinical relevance of anti-Sp100 antibodies in PBC has been extensively studied in recent years, but a correlation between levels of anti-PML or anti-Sp140 and PBC was studied much less frequently and has still not been fully defined [8,17,21,22,32,33].

The aim of this study was to analyze the immune response against Sp140, Sp100 and PML proteins in a well-characterized group of PBC patients from Poland. We evaluated the occurrence, frequency, specificity, and diagnostic significance of anti-sp140 antibodies, anti-Sp100 and anti-PML antibodies and compared the results with biochemical and histological parameters.

## 2. Materials and Methods

### 2.1. Patients

Serum samples were collected from PBC patients treated at the Centre of Postgraduate Medical Education in Warsaw, Poland. The diagnosis of PBC was established using the practical guidelines of the European Association for the Study of the Liver for PBC [34]. A biopsy was performed in all of the patients and the results were divided into four groups reflecting Ludwig’s staging. Patients were grouped according to their fibrosis level: group I/II (early stage) patients with F1 and F2 fibrosis and group III/IV (advanced stage) included patients with F3 and F4 fibrosis. Four patients presented ambiguous histological stage. Patients with positive serum levels for hepatitis B surface antigen, anti-hepatitis A and hepatitis C virus, patients with alcoholism and AIH/PBC overlap syndrome were excluded from the study. Seventeen PBC patients were classified as AMA-negative. Control samples were collected from patients with autoimmune hepatitis–AIH, primary sclerosing cholangitis–PSC and rheumatoid arthritis–RA. Additional serum samples were collected from healthy adult blood donors at the Warsaw Blood Bank. AIH was established based on characteristic clinical signs and symptoms, laboratory anomalies (elevated serum aspartate aminotransferase [AST] or alanine aminotransferase [ALT], and increased serum total IgG concentration) and the presence of one or more characteristic autoantibodies (ANA, anti-smooth muscle antibodies, anti-liver-kidney microsomal antibody type 1, or anti-liver cytosol type 1), and histological findings. Other conditions that can cause chronic hepatitis were excluded, such as viral, hereditary, metabolic, cholestatic, and drug-induced diseases. The Revised Original Scoring System of the International Autoimmune Hepatitis Group was used for assessment of AIH. PSC was established using magnetic resonance cholangiopancreatography or endoscopic retrograde cholangiopancreatography. The study protocol was conducted in accordance with the ethical guidelines of the Declaration of Helsinki and was approved by the Ethical Committee of the Centre of Postgraduate Medical Education, Warsaw (approval 12 June 2019, number 71/PB/2019). Informed consent was obtained from all patients. All samples were frozen at −80 °C and tested immediately after defrosting.

### 2.2. Methods

#### 2.2.1. ELISA “In House” Technique for Anti-Sp140 Detection

The human recombinant protein Sp140 (*E. coli*) was obtained from the Abnova Corporation (Taipei City, Taiwan).

Anti-Sp140 was determined using an in-house enzyme-linked immunosorbent assay (ELISA) developed at the Department of Biochemistry and Molecular Biology, Centre of Postgraduate Medical Education. Wells of flat-bottom microtiter plates (Costar, Corning, NY USA) were coated overnight with 0.1 mL of 1 μg/mL solution containing Sp140 protein in 0.1 M bicarbonate buffer (pH 9.9) at 4 °C, then saturated with 1% bovine serum albumin (BSA) in phosphate buffered saline pH 7.4 (PBS) and washed with PBS-0.1% Tween (PBS-Tween). The tested sera (1:100 dilution) were incubated in coated plates for 1 h at room temperature in PBS-Tween with 0.5% BSA. The plates were washed, incubated for 1 h at room temperature with 0.1 mL of horseradish peroxidase-conjugated human IgG antibodies (Daco A/S Denmark; dilution 1:50,000 in PBS-Tween), and washed again. A color reaction was developed by adding 0.1 mL of tetramethylbenzidine (TMB, Serva, Heidelberg, Germany) for 15 min and stopping the reaction with 0.5 M H_2_SO_4_. The absorbance was measured at 450 nm with an automatic plate reader (Multiscan RC, Labsystem, Vantaa, Finland).

The quality and repeatability of the test was assessed by examining 60 serum samples (30 positive samples from patients with PBC and 30 blood donors), each in 3-plicate and 21 technical repeats.

The final levels of antibodies were calculated with reference to our standard serum, which had been diluted to five different concentrations (10, 30, 50, 200 and 500 units/mL). Results lower than 30 units/mL were arbitrarily determined as negative. Results in the range of 30–50 units/mL were recognized as slightly positive. Results above 50 units/mL were considered as positive. The intra-assay performance of our ELISA “in-house” test was 4.6% and the inter-assay coefficient of variation was 11.1%.

#### 2.2.2. Detection of Anti-Sp100, Anti-PML Antibodies, and AMA M2

Anti-Sp100, anti-PML, and AMA M2 antibodies were determined using a commercially available kit according to the manufacturer’s instruction: anti-Sp100-ELISA kit (Imtec-Human; Wiesbaden, Germany); anti-PML antibodies-Euroline Test System–Profile Autoimmune Liver Diseases (Euroimmun, Lubeck, Germany), AMA M2-kit AMA M2-3E (Euroimmun, Lubeck, Germany). The levels of AMA M2 and anti-Sp100 antibodies were calculated in U/mL, results for anti-PML antibodies were evaluated with EUROLineScan and presented in EUROLineScan units.

### 2.3. Clinical Statistics

Calculations were done with Stata Statistical Software v. 6.0 (Stat-Soft, Cracow, Poland) and MedCalc for Windows, version 7.4.1.0 (MedCalc Software, Mariakerke, Belgium).

Results were summarized using standard methods: mean and standard deviation for continuous variables and frequency tables for categorical variables. Comparisons between patients positive and negative for anti-Sp100, anti-PML, and anti-Sp140 were made using the Student’s t-test or Kruskal-Wallis test for continuous variables and the χ^2^ test for categorical variables. The multivariate proportional-hazards regression model was used to investigate survival. The proportional hazard assumption was checked using tests based on Schoenfeld residuals. Survival curves were estimated using the Kaplan–Meier method and compared with the log-rank test. All analyses were two-sided, and *p*-value less than 0.05 was considered as statistically significant.

We calculated sensitivity, specificity, positive and negative predictive values (PPV, NPV), and positive and negative likelihood ratios (LR+, LR−) according to statistical criteria.

Statistical analysis of the ROC curve was performed using Prism software (GraphPad, La Jolla, CA, USA).

## 3. Results

### 3.1. Clinical, Histological, and Laboratory Features of PBC Patients and Control Groups

The clinical, histological, and laboratory features of PBC patients and control groups are summarized in Table 1.

Activity of AST and ALT was elevated in 75% and in 65% of PBC patients, respectively. Over 75% of patients presented increased activity of AP and γ-GT, while a decreased level of albumin was determined in 45% of PBC patients. The total bilirubin was higher in 85% of samples. AMA was detected in 82% of patients’ serum.

### 3.2. Occurrence and Diagnostic Accuracy for Antibodies Directed against Components of Promyelocytic Leukemia Protein Nuclear Bodies in PBC

**Figure 1:**
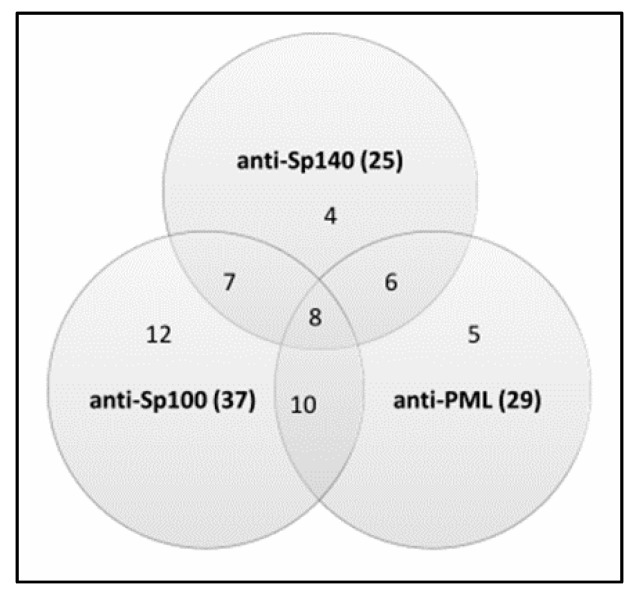
The frequency of responses against nuclear body components Sp140, Sp100, and PML in 93 PBC patients.

In general, 52 (56%) PBC patients had at least one of three anti-PML body reactivities. We observed an autoimmune reaction against multiple nuclear components in some PBC patients (Figure 1).

Anti-Sp140 autoantibodies were found in 25 (27%) out of 93 PBC individuals. Anti-Sp100 and anti-PML antibodies were found in 37 (40%) and 29 (31%) PBC patients, respectively. In the control group, among PSC patients, we detected anti-Sp140 antibodies in one (4%) patient and anti-Sp100 in 3 (12%) patients. Among RA patients, we determined anti-Sp100 antibodies in only one (5%) sample. Anti-PML antibodies were found only in the PBC cohort. The examined antibodies were not found in any of the healthy controls.

Specificity for these antibodies detected by ELISA was: 98.8%, 95.3%, and 100% for anti-Sp140, anti-Sp100, and anti-PML, respectively. The positive and negative likelihood ratios were determined from suitable sensitivities and specificities (Table 2).

Data in Table 2 presents positive and negative predictive values for anti-Sp140, anti-Sp100, and anti-PML autoantibodies in serum of PBC patients. The proportion of patients with positive test results who were correctly diagnosed–Positive Predictive Value (PPV) showed a very high value for all the tested antibodies, the highest for the anti-PML antibody (100%). The proportion of patients with negative test results, who were correctly diagnosed-Negative Predictive Value (NPV), presented the highest value for anti-Sp100 (59%).

**Figure 2:**
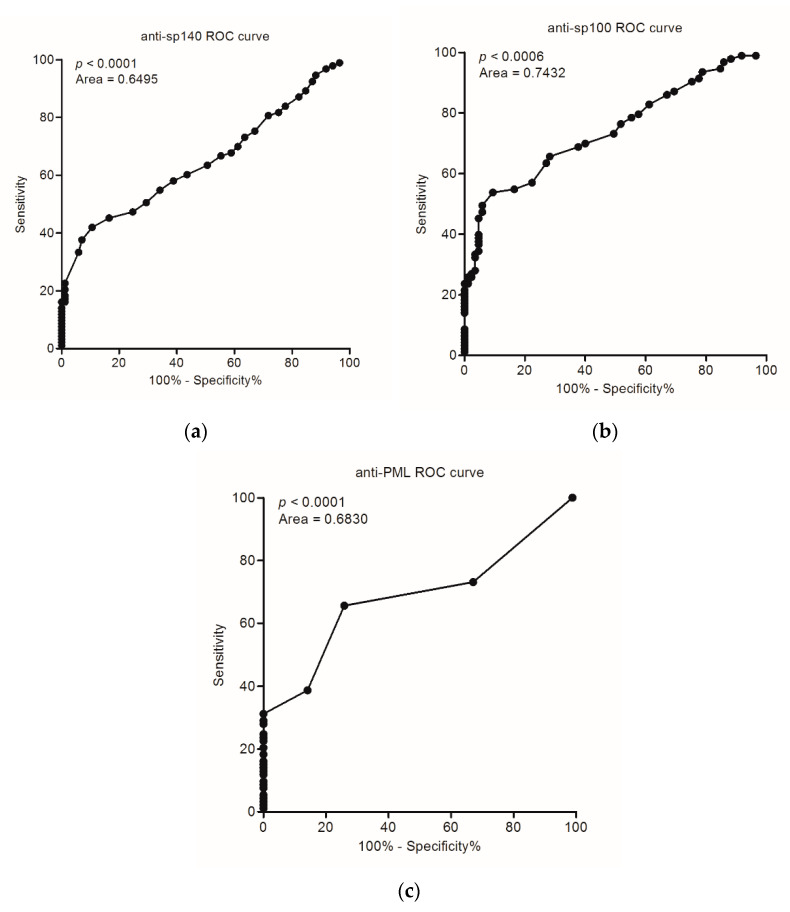
ROC curve analysis for serological detection of PBC (**a**) anti-Sp140, (**b**) anti-Sp100, and (**c**) anti-PML.

The accuracy of the tests detecting these antibodies and their ability to differentiate PBC patients and healthy cases was 61%, 66%, and 64% for anti-Sp140, anti-Sp100, and anti-PML, respectively, and disease prevalence was 52% for each antibody. In our study, the area under the receiver operating characteristics (ROC) curve was greatest for anti-Sp100 testing (0.7432, *p* < 0.0001). ROC curve analysis for serological detection of PBC anti-Sp140, anti-Sp100, and anti-PML is shown in Figure 2, panels (a), (b), and (c), respectively.

Analysis showed that the positive detection rate of PBC was 56% in the combined detection of anti-Sp140, anti-Sp100, and anti-PML antibody, compared with 27% in single anti-Sp140, 40% in single anti-Sp100, and 31% in single anti-PML antibody detection; thus, the diagnosis rate was increased by about 20%, and the difference was statistically significant, *p* < 0.05 (Table 2). The accuracy of the combined detection of these antibodies and their ability to differentiate PBC patients and healthy cases slightly increases to 72%.

**Figure 3:**
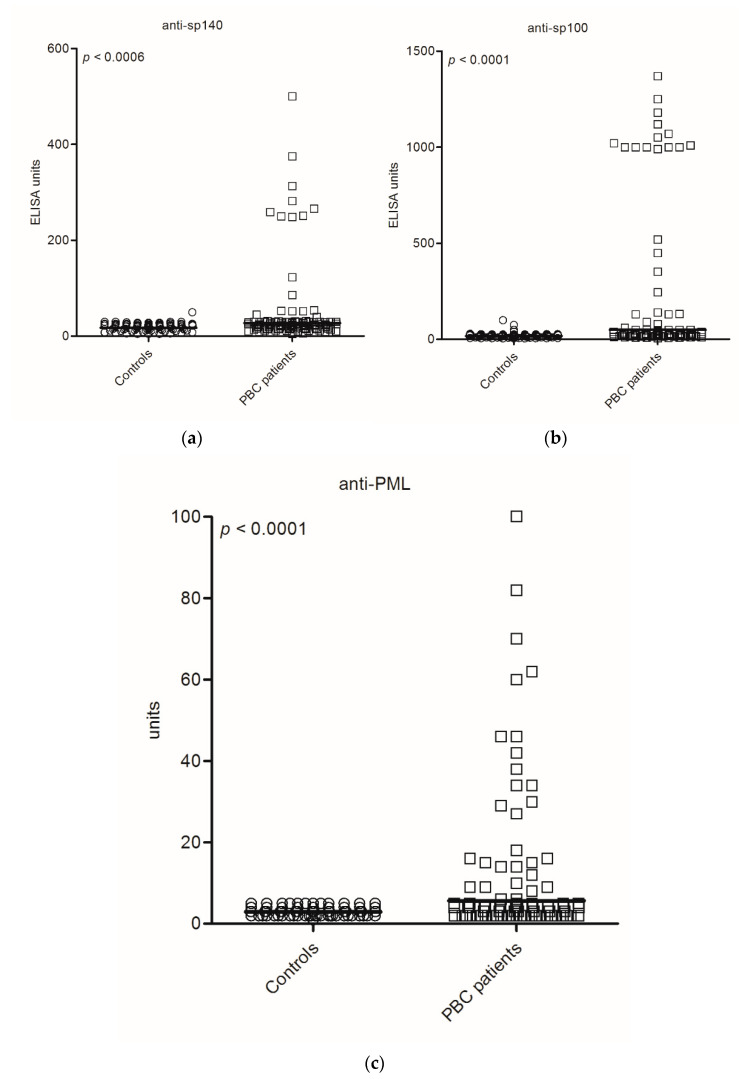
Distribution of: anti-Sp140 antibodies (**a**), anti-Sp100 antibodies (**b**), anti-PML antibodies, (**c**) in PBC patients and the control group.

The levels of anti-PML NB protein antibodies in sera of PBC patients and the control group are presented in Figure 3.

The level of anti-Sp140 antibodies is shown in Figure 3a. Over 40% of PBC patients demonstrated enhanced levels of anti-Sp140 antibodies (>100 U/mL). The level of anti-Sp100 antibodies is shown in Figure 3b, 22% of anti-Sp100 positive PBC patients demonstrated a high level, above 400 U/mL. The level of anti-PML antibodies was presented in Figure 3c, where a high level above 30 units was determined in 58% of anti-PML-positive PBC patients.

We found a significant difference between the levels of antibodies in PBC patients and the control group: 52 U/mL vs. 19 U/mL, *p* < 0.0006 for anti-Sp140 antibodies, 210 U/mL vs. 20 U/mL, *p* < 0.0001 for anti-Sp100 antibodies and 15 U/mL vs. 3.1 U/mL, *p* < 0.0001 for anti-PML. A correlation between levels of anti-Sp140, anti-Sp100 and anti-PML was observed: anti-Sp140 vs. anti-Sp100–R = 0.80, *p* < 0.001; anti-Sp140 vs. anti-PML–R = 0.51, *p* < 0.001; anti-Sp100 vs. anti-PML–R = 0.59, *p* < 0.001.

We also compared the presence and level of serum anti-PML NB antibodies with the presence of AMA-M2 in PBC patients. Anti-Sp140 antibodies were identified in 41% of AMA-M2-negative vs. 24% in AMA-M2-positive samples, anti-Sp100 were detected in 29% of AMA-M2-negative vs. 42% in AMA-M2 positive patients and anti-PML in 35% AMA-M2-negative vs. 30% in AMA-M-2-positive patients. These differences were not statistically significant, but anti-PML, and especially anti-Sp140 antibodies, were more common in the AMA-M2-negative group of PBC patients. On the contrary, anti-Sp100 antibodies were recognized more often in the AMA-M2-positive group. Among the 17 AMA-negative patients, 4 (24%) patients were positive for all three reactivities (Sp140, Sp100, PML) and 3 (18%) patients were positive for at least two activities. In addition, among the 76 AMA-positive patients, 4 (5%) were positive for all three antibodies: anti-Sp140, anti-Sp100, and anti-PML, while 20 patients (26%) were positive for at least two activities.

**Figure 4:**
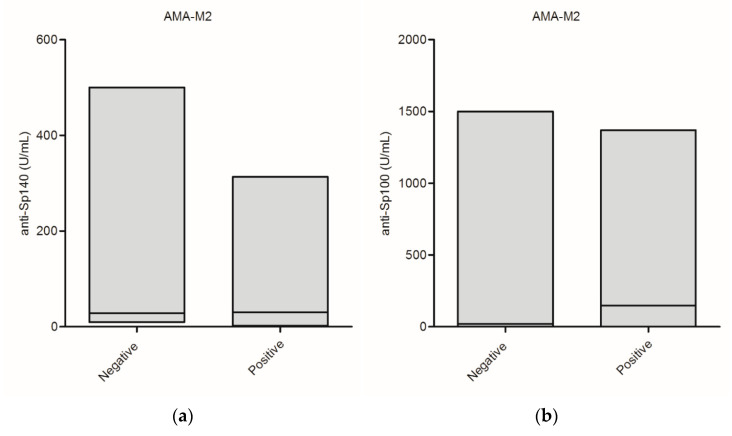

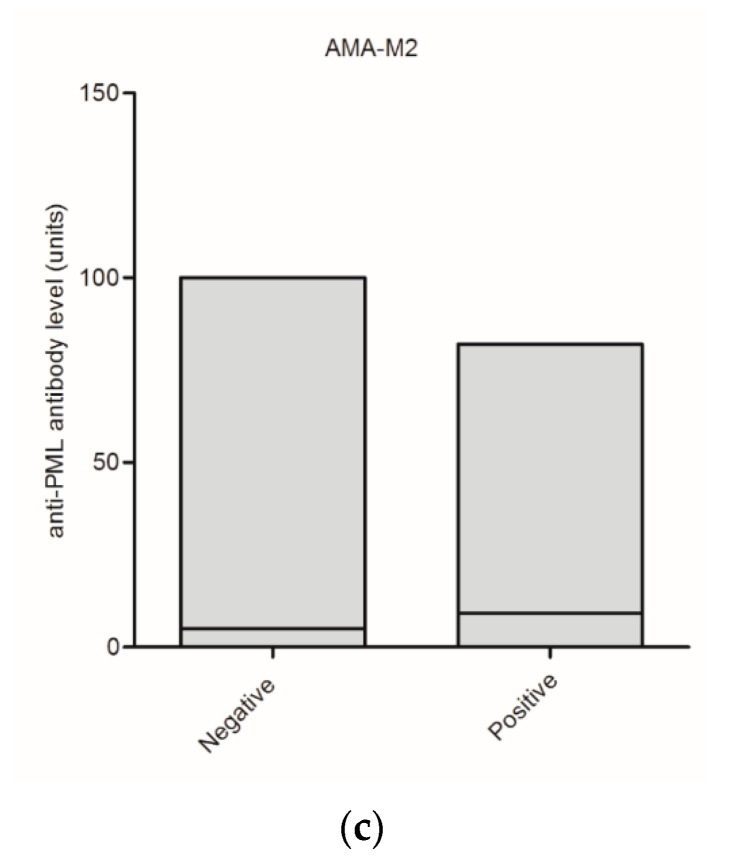
Serum levels of anti-promyelocytic leukemia nuclear body components antibodies in AMA-positive and AMA-negative patients: (**a**) anti-Sp140 antibodies, (**b**) anti-Sp100 antibodies, and (**c**) anti-PML antibodies.

Serum levels of anti-PML NB antibodies in AMA-positive and AMA-negative patients are summarized in Figure 4. Figure 4 presents serum level of: anti-Sp140 antibodies-panel (a), anti-Sp100 antibodies-panel (b), and anti-PML antibodies-panel (c).

No significant difference between the concentration of anti-Sp100 autoantibodies and AMA-positive or negative status was observed (371.3 ± 543.2 vs. 178.4 ± 353.4; *p* = 0.07). Nevertheless, the mean levels of the anti-Sp140 and anti-PML antibodies in AMA-positive and AMA-negative groups differed significantly (33.0 ± 50.8 vs. 143.7 ± 154.5; *p* = 0.001 and 9.1 ± 23.0; *p* = 0.005).

Combining anti-Sp140, anti-Sp100, and anti-PML is useful to help diagnosis for the case of AMA negative (Table 2). Positive detection rate of PBC AMA negative was 65% in the combined detection of anti-Sp140, anti-Sp100, and anti-PML antibody, compared with 41% in single anti-Sp140, 29% in anti-Sp100, and 35% in single anti-PML antibody detection in this group; thus, the diagnosis rate was increased about 25–30%, and the difference was statistically significant primarily for anti-Sp100 antibody, *p* = 0.038. The accuracy of the combined detection of these anti-nuclear antibodies in AMA negative cases increased to 90%. We evaluated the diagnosability by a combination of four factors anti-Sp140, anti-Sp100 and anti-PML together with AMA. Sensitivity increased significantly from 82% in single AMA detection to 94% with difference being statistically significant, *p* = 0.012 and accuracy increased a little from 88% to 94%.

### 3.3. Biochemical Features of Patients with PBC According to the Status of Anti-PML NB Antibodies

A comparison of biochemical parameters in the groups of anti-PML NB positive and negative PBC patients showed similar results, with the exception of total bilirubin and alkaline phosphatase. Patients with positive reactivity for anti-PML NB antibodies (positive for at least one of three anti-PML body reactivities: anti-Sp140, anti-Sp100, anti-PML) showed a higher concentration of total bilirubin (2.8 vs. 1.6, *p*= 0.045) and a higher concentration of alkaline phosphatase (559.0 vs. 406.5, *p* = 0.040). Biochemical results at the time of diagnosis in 93 patients with PBC according to each anti-Sp140, anti-Sp100, and anti-PML antibody type status are presented in Table 3.

### 3.4. Autoantibodies Directed against PML NBs and the Survival of Patients

**Figure 5:**
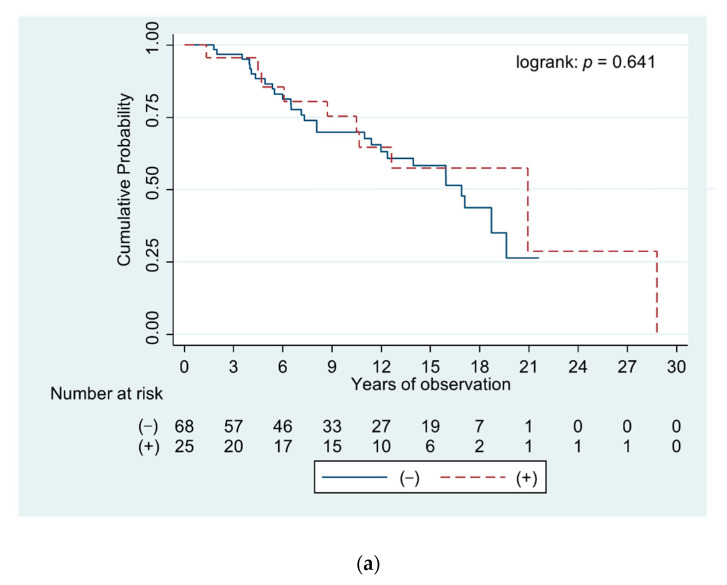

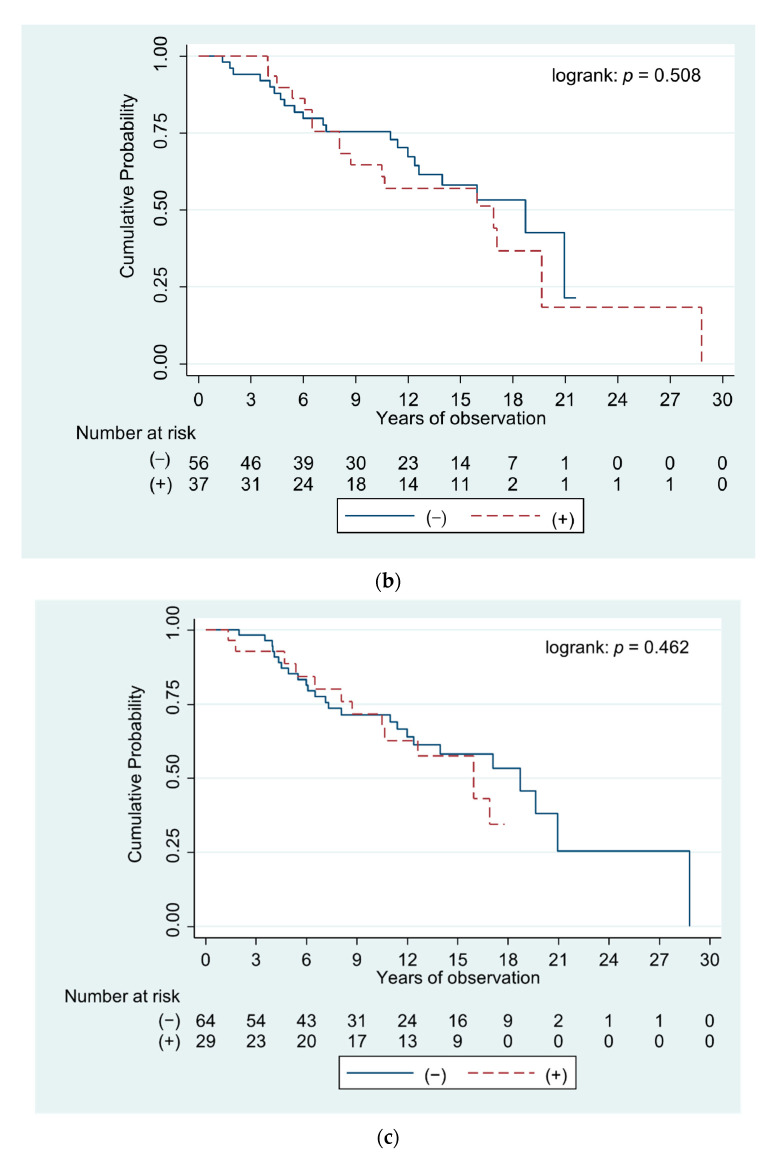
Curves demonstrating survival of PBC patients positive and negative for specific antibodies directed against components of promyelocytic leukemia protein nuclear bodies: (**a**) anti-Sp140 antibodies, (**b**) anti-Sp100 antibodies, and (**c**) anti-PML antibodies.

Analysis of the survival of patients positive and negative for anti-PML NB components (Figure 5) demonstrated that presence of these autoantibodies did not correlate with the length of life or time to liver transplantation in PBC patients.

The curves demonstrate survival of PBC patients positive and negative for specific antibodies directed against Sp140, Sp100, and PML proteins–Figure 5a–c, respectively.

The survival time of patients or the period for liver transplantation was shortest for people with anti-PML antibodies, although it was not statistically significant.

It is interesting that among patients with presence of two or three types of anti-PML NB antibodies, deaths or the need for transplantation were more frequent than in the group of patients without these antibodies. In a group of 31 PBC patients with at least two types of these antibodies, we observed 18 negative events (58%): 8 deaths and 10 transplantation, in group of 41 patients without anti-PML NB antibodies only 9 negative events (22%). An odds ratio (OR), measured for these two group was 4.9 [95% CI: 1.8–13.8, *p* = 0.002].

### 3.5. Biochemical Parameters and the Survival of Patients

The results of laboratory tests performed at the time of diagnosis were comparable in patients positive and negative for the analyzed autoantibodies, with the exception of total bilirubin and alkaline phosphatase concentrations. Multivariate proportional hazard’s regression model did not demonstrate an increased risk of death for patients with a higher alkaline phosphatase concentration. Serum total bilirubin was the only independent predictor of poor prognosis in PBC (Table 4).

Patients with an elevated level of total bilirubin (>1.1 mg/dL at the time of diagnosis) had a 5.7 [95% CI, 2.7–12.3] times higher risk of death than patients with a normal bilirubin concentration.

### 3.6. Autoantibodies Directed against PML NBs and Histological Parameters

We compared the presence of the examined antibodies between patients with early (I/II) and advanced (III/IV) histological stages of PBC. Among 52 PBC patients with early histological stages (I/II) of the disease, according to Ludwig’s classification, 25 (48%) were anti-PML NB positive. In the group of PBC patients with advanced histological stages (III/IV), 27 out of 37 (70%) were anti-PML NB positive (*p* = 0.039).

## 4. Discussion

Some studies have reported that nuclear body proteins are important targets of ANA in PBC patients [17,18,19]. The study performed by Granito et al. highlighted the significance of anti-Sp140 [31]. Data from Western and Southern Europe also confirmed the importance of anti-Sp100 and anti-PML specific antibodies in PBC patients [17,21,24,27,33], similarly to studies from North America [23]. Meta-analyses evaluating the diagnostic accuracy of anti-Sp100 antibodies showed high specificity (but low sensitivity), with the possibility of using them as biomarkers in PBC [19,28]. The sensitivities for anti-Sp100 or anti-PML, as well as anti-Sp140 detected in our study are also low, despite being highly specific for PBC. Therefore, their prognostic role might be divisive, which stays in accordance with other detailed reports describing the occurrence of anti-promyelocytic leukemia nuclear body components [21,31]. In addition, our previous study [33] confirmed the very high specificity of anti-Sp100 antibodies, but their relationship with biochemical parameters, survival of patients and the histological picture was not investigated. The presented work further analyzed the immune response against Sp100, as well as Sp140 and PML proteins in PBC patients and combined the data with biochemical and histological parameters. Anti-Sp100 and anti-PML antibodies were screened using a commercial kit. However, anti-Sp140 antibodies were detected using the ELISA “in house” method (using a recombinant protein), which presents very high specificity. The developed quantitative test is easy to perform and capable of identifying even small differences in the autoantibody titer among PBC patients. Moreover, it may detect lower-titer or low-avidity antibodies. The ability of the developed ELISA technique to detect anti-Sp140 and other antinuclear antibodies was also evaluated in the light of PBC clinical diagnoses. It has been demonstrated that recombinant anti-Sp140 may be a useful tool in future serum tests of patients with PBC.

We observed that autoantibodies directed against components of PML NBs are highly specific for PBC and they were present in 52% of PBC patients, including AMA-negative. In contrast, anti-PML NBs were found in only 6% of the controls (none in the healthy control group).

The measured sensitivity of anti-Sp140 for PBC was 27%, anti-Sp100 40%, and anti-PML 31%, which was higher than the values previously reported [21,31]. The specificity and PPV of these antibodies for PBC patients was very high, 95–100% and 90–100%, respectively. The calculated LR+ value showed that positive test results for anti-Sp140 antibodies in the studied cohort were about 23 times as likely to be obtained in an individual with PBC than in someone without the disorder. The observed very high LR+ (22.9) indicates that the test may be used for diagnosis of PBC. A slightly higher prevalence of anti-Sp140 and anti-PML in AMA-negative PBC patients was also observed, but was not statistically significant. Granito and co-workers also found anti-Sp140, anti-PML, and anti-Sp100 antibodies were more frequent in AMA-negative patients than in AMA-positive [31]. On the contrary, our study revealed that anti-Sp100 antibodies were detected more often in the AMA-M2 positive group. Correspondingly to the published reports [21,31], the frequent coexistence of anti-Sp140, anti-Sp100 and anti-PML antibodies suggests an autoimmune reaction against multiple nuclear body components in some PBC patients. We presented a distribution of anti-Sp140, anti-Sp100, and anti-PML antibodies in PBC patients and the control group. The study performed by Mytilinaiou et al. also showed the anti-PML and anti-Sp100 titers in the screened 150 AMA-positive PBC patients [21]. We also detected the presence of these antibodies in both groups of PBC patients–AMA-M2 positive and negative. A much higher level of anti-Sp140 and anti-PML antibodies in the AMA-M2 negative group was observed.

We evaluated the connection between anti-Sp140, anti-Sp100 and anti-PML with the clinical outcome of PBC. Mytilinaiou and co-workers analyzed clinical, immunological, and histological data of 170 PBC patients in relation to the presence of anti-Sp100 and anti-PML determined by the indirect immunofluorescence technique and line immunoassay. They suggested an association of anti-Sp100 levels with the Mayo risk score and a correlation between double anti-PML/Sp100 reactive cases [21]. Similarly, our study revealed that among PBC patients positive for anti-Sp100, or anti-PML, as well as for anti-Sp140, cases with advanced histological stages are much more common, especially in AMA-negative patients. There was no direct association between the presence of anti-Sp140, anti-Sp100 and anti-PML antibodies, and the initial onset or significantly shorter survival of PBC patients. However, in the group of patients with at least two types of these antibodies, deaths or the need for transplantation were more frequently observed than in the group of patients without these antibodies. The presence of two or three of these autoantibodies reveled a subclass of PBC patients with more progressive liver disease, in comparison to those who had no reactivity to these antigens. Mytilinaiou and co-workers compared other, biochemical and clinical, parameters between positive and negative groups and they did not reveal significant differences [21]. Contrary to these results, we found an association between positive reactivity of autoantibodies directed against components of PML nuclear bodies and higher concentrations of total bilirubin and alkaline phosphatase.

In our work, the multivariate Cox analysis was performed in the whole group of PBC patients, and this model fits well with several clinical, biochemical, and serological variables. Such an attempt clearly showed that the main risk factor in our patients was serum total bilirubin. A similar conclusion was made several years ago based on data from 4845 patients included in 15 North American and European long-term follow-up cohort studies. The authors suggested that levels of alkaline phosphatase and bilirubin can predict outcomes (liver transplantation or death) of patients with PBC and might be used as surrogate end points in therapy trials [35]. Furthermore, Perez et al. suggested that normal bilirubin and ALP levels are associated with the lowest risk for liver transplantation or death in patients with PBC [36].

In conclusion, our study on a well-diagnosed group of PBC patients confirmed very high specificity for anti-Sp140, anti-Sp100, and anti-PML antibodies. We propose that autoantibodies directed against components of PML nuclear bodies may aid in serologic diagnoses, also in cases in which anti-mitochondrial antibodies are not detectable. These antibodies in patients with cholestasis would facilitate the diagnosis of AMA-negative PBC. Testing for these autoantibodies would help to define their clinical and pathogenic significance, as their potential pathogenic role has not been studied yet. We agree with Terziroli Beretta-Piccoli and coworkers [1] that it is necessary to study the mechanisms causing the production of anti-nuclear antibodies and identify the role of T cell, which distinguish the antigenic target of PBC specific antinuclear antibodies. It could be very interesting as well to investigate homologies with microbial proteins. Overall, understanding the link between propagation patterns of antibodies, including immunoglobulins directed against PML NBs, and the pathogenesis of autoimmune diseases may support the development of diagnostic tools for early detection of PBC.

## Figures and Tables

**Table 1:** The demographic, biochemical, immunological, and histological features of PBC patients and control groups.

	Primary Biliary Cholangitis Patients (*n* = 93)	Autoimmune Hepatitis Patients (*n* = 10)	Primary Sclerosing Cholangitis Patients (*n* = 25)	Rheumatoid Arthritis Patients (*n* = 20)	Healthy Adult Blood Donors (*n* = 20)
Age, years	51 (25–68)	49 (20–70)	48 (20–65)	40 (24–67)	32 (20–55)
Females/males	89/4	8/2	11/14	20/0	15/5
Bilirubin (Total), mg/dL	2.4 (2.4)	2.2 (1.9)	1.5 (2.8)	0.9 (0.7)	0.9 (0.3)
AST, U/L	83.9 (51.2)	43 (73)	99 (67)	19 (27)	24 (24)
ALT, U/L	93.6 (72.5)	60 (50)	87 (64)	26 (38)	15 (30)
AP, U/L	506.5 (429.2)	222 (173)	344 (225)	44 (28)	40 (20)
γ-GT, U/L	334.3 (291.9)	230 (206)	350 (250)	54 (59)	20 (5)
AMA or M2 antibody Anti-Sp100 antibody Anti-Sp140 antibody Anti-PML antibody	76 (82%) 37 (40%) 25 (27%) 29 (31%)	0 (0%) 0 (0%) 0 (0%) 0 (0%)	0 (0%) 3 (12%) 1 (4%) 0 (0%)	0 (0%) 1 (5%) 0 (0%) 0 (0%)	0 (0%) 0 (0%) 0 (0%) 0 (0%)
Early histological stage (I/II)	52 (56%)	6 (60%)	11 (44%)	0	0
Advanced histological stage (III/IV)	37 (40%)	3 (30%)	5 (20%)	0	0
Ambiguous histological stage	4 (4%)	0	0	0	0

Data are presented as mean (SD). Abbreviations: γ-GT, γ-glutamyl transpeptidase; ALT, alanine aminotransferase; AP, alkaline phosphatase; AST, aspartate aminotransferase; Normal value: bilirubin < 1.2 mg/dL; AST < 40 U/L; ALT < 40 U/L; AP < 115U/mg/dL; γ-GT < 50 U/L; albumin 3.5–5.5 g/dL, γ-globulin < 3 g/dL. Conversion factors to SI units are as follows: for bilirubin, 17.1; for AST, ALT, AP and γ-GT, 0.0167.

**Table 2:** Summary of sensitivities, specificities, positive and negative predictive values, likelihood ratios and AUC for anti-Sp140, anti-Sp-100 and anti-PML antibodies in patients with PBC.

	Sensitivity % (95% CI)	Specificity % (95% CI)	Positive Predictive Value (PPV) % (95% CI)	Negative Predictive Value (NPV) % (95% CI)	Positive Likelihood Ratio (LR+)	Negative Likelihood Ratio (LR−)	AUC
Anti-Sp140	27 (18–39)	99 (94–100)	96 (79–99)	55 (51–67)	22.9	0.7	0.6495
Anti-Sp100	40 (24–45)	95 (88–99)	90 (70–96)	59 (52–69)	8.5	0.6	0.7432
Anti-PML	31 (25–47)	100 (99–100)	100 (92–100)	57 (53–70)	–	0.7	0.6830
Combined detection: anti-Sp140, anti-Sp100, anti-PML	56 (45–66)	95 (88–99)	92 (82–97)	64 (59–69)	11.0	0.5	
Combined detection: anti-Sp140, anti-Sp100, anti-PML (AMA negative)	65 (38–86)	95 (88–99)	73 (50–88)	93 (88–96)	13.8	0.4	
Combined detection: anti-Sp140, anti-Sp100, anti-PML (AMA positive)	94 (86–98)	95 (88–99)	96 (89–98)	93 (86–98)	19.9	0.1	

Abbreviations: AUC, area under curve.

**Table 3:** Biochemical results at the time of diagnosis in 93 patients with PBC according to each anti-Sp140, anti-Sp100, and anti-PML antibody type status.

	Anti-Sp140 Antibody			Anti-Sp100 Antibody			Anti-PML Antibody		
	Positive *n* = 25	Negative *n* = 68	*p*	Positive *n* = 37	Negative *n* = 56	*p*	Positive *n* = 29	Negative *n* = 64	*p*
Bilirubin (total), mg/dL	3.0 (3.5)	1.7 (1.6)	0.016	2.5 (3.2)	1.5 (1.4)	0.037	2.8 (3.4)	1.5 (1.4)	0.010
AST, U/L	108.8 (95.0)	72.5 (35.0)	0.008	86.9 (55.4)	68.8 (52.8)	ns	90.7 (48.5)	74.3 (39.0)	ns
ALT, U/L	98.0 (95.4)	88.1 (75.1)	ns	95.3 (94.5)	89.1 (69.8)	ns	105.3 (96.2)	87.4 (70.8)	ns
AP, U/L	586.7 (530.9)	398.9 (304,1)	0.036	557.3 (466.6)	429.8 (362.1)	ns	544.5 (458.6)	392.3 (285.1)	0.048
γ-GT, U/L	367.2 (345.8)	329.9 (327.7)	ns	343.4 (317.5)	310.8 (291.0)	ns	341.3 (337.7)	300.9 (301,6)	ns

Data are presented as mean (SD). Abbreviations: γ-GT, γ-glutamyl transpeptidase; ALT, alanine aminotransferase; AP, alkaline phosphatase; AST, aspartate aminotransferase; Normal value: bilirubin < 1.2 mg/dL; AST < 40 U/L; ALT < 40 U/L; AP < 115U/mg/dL; γ-GT < 50 U/L; albumin 3.5–5.5 g/dL, γ-globulin < 3 g/dL. Conversion factors to SI units are as follows: for bilirubin, 17.1; for AST, ALT, AP and γ-GT, 0.0167.

**Table 4:** Cox’s proportional hazard regression for analysis of factors associated with overall survival in 93 patients with primary biliary cirrhosis.

Variable	*p*
Age at disease onset (>51 years)	>0.1
Bilirubin (Total) (>1.1 mg/dl)	<0.001 *
AST (>71 IU/l)	0.096
ALT (>76 IU/l)	>0.1
AP (>458 IU/l)	>0.1
γ-GT (>260 IU/l)	>0.1

Conversion factors to SI units are as follows: for bilirubin, 17.1; for AST, ALT, AP and γ-GT, 0.0167. Among the 93 patients included in the study, 20 deaths related to liver disease were reported and 18 patients required/underwent liver transplantation. Continuous variables were categorized using median (values) as a cut-off (value in brackets). * An elevated level of serum total bilirubin at the time of diagnosis was the only significant independent factor in a poor prognosis; hazard ratio 5.7; 95% CI, 2.7–12.3.

## Data Availability

All available data are presented within the article or are available on request from the corresponding author.
